# Study design and statistics in the epidemiology of breast cancer

**DOI:** 10.1038/sj.bjc.6603370

**Published:** 2006-10-03

**Authors:** L Maraqa, M Lansdown

**Affiliations:** 1 Breast Unit, Lincoln Wing, St James's University Hospital, Beckett Street, Leeds LS9 7TF, UK

**Sir**,

Your recent article by García-Closas *et al* (2006) raises interesting discussion points regarding tumour behaviour in Poland. If correct, these results may have implications for health care in Poland, but we disagree that the findings can be generalised to other populations, particularly when the reported ER negativity was 41%. There were several flaws in the study design and analysis that make such a conclusion unsafe. Firstly, we understand mammographic screening is not routine in Poland (reflected in the low incidence (6%) of DCIS). The authors should have made clear whether mammography was in fact offered to equal numbers in the study and control groups. It is possible that the results were biased by differential access to and uptake of mammography between the groups; perhaps, better educated women were both more likely to take HRT and seek mammograms. Indeed, the absolute differences for better education between the two arms (10%, unadjusted OR=1.89) and history of a previous mammogram (8%, unadjusted OR=1.39) support this suggestion.

Another potential error lies in the histology. The rate of agreement between the original Polish and the subsequent American analysis varied from 18 to 80%, depending on tissue type. However, nearly one in five (*n*=428) did not benefit from an American review, yet were still included in the study. With such high rates of discordance, it was hazardous to assume the original diagnosis was correct in the nonreviewed group. If the ratios for error are the same as the reviewed group, then at least 4.9% of all samples would have been misclassified, thus challenging many of the statistical results (see Table 1).

Analysis of data from studies of this kind is fraught with difficulties and such statistical methods are not always illuminating. We believe there is a great potential for over interpretation.

## Figures and Tables

**Table 1:** Analysis of potential misclassification in nonreviewed histology

	**% of reviewed histology**	**Reported discordance (%)**	**Projected number of nonreviewed cases**	**Projected misclassification in nonreviewed cases**	**Proportion to total sample (%)**
Ductal	58	20	428*58%	248*20%	2.2
			248 cases	50 cases	
Lobular	16	32	428*16%	68*32%	0.9
			69 cases	22 cases	
Mixed	12	82	428*12%	51*82%	1.8
			51 cases	42 cases	
Other	14	No data: Assume=0	428*14%		—
			60 cases	0 cases	
					
Total	100	—	428 cases	114 cases	4.9

## References

[bib1] García-Closas M, Brinton LA, Lissowska J, Chatterjee N, Peplonska B, Anderson WF, Szeszenia-Dabrowska N, Bardin-Mikolajczak A, Zatonski W, Blair A, Kalaylioglu Z, Rymkiewicz G, Mazepa-Sikora D, Kordek R, Lukaszek S, Sherman ME (2006) Established breast cancer risk factors by clinically important tumour characteristics. Br J Cancer 95: 123–129, doi:10.1038/sj.bjc.6603207 16755295 10.1038/sj.bjc.6603207 PMC2360503

